# Class 3 semaphorins expression and association with innervation and angiogenesis within the degenerate human intervertebral disc

**DOI:** 10.18632/oncotarget.4274

**Published:** 2015-06-15

**Authors:** Abbie L. A. Binch, Ashley A. Cole, Lee M. Breakwell, Anthony L. R. Michael, Neil Chiverton, Laura B. Creemers, Alison K. Cross, Christine L. Le Maitre

**Affiliations:** ^1^ Sheffield Hallam University, Sheffield, South Yorkshire, United Kingdom; ^2^ Sheffield Teaching Hospitals, Sheffield, South Yorkshire, United Kingdom; ^3^ Universitair Medisch Centrum, Orthopaedics Department, Utrecht, Netherlands

**Keywords:** Pathology Section, intervertebral disc, cytokines, semaphorins, innervation, angiogenesis

## Abstract

Nerve and blood vessel ingrowth during intervertebral disc degeneration, is thought to be a major cause of low back pain, however the regulation of this process is poorly understood. Here, we investigated the expression and regulation of a subclass of axonal guidance molecules known as the class 3 semaphorins, and their receptors; plexins and neuropilins within human NP tissue and their regulation by pro-inflammatory cytokines. Importantly this determined whether semaphorin expression was associated with the presence of nerves and blood vessels in tissues from human intervertebral discs. The study demonstrated that semaphorin3A, 3C, 3D, 3E and 3F and their receptors were expressed by native NP cells and further demonstrated their expression was regulated by IL-1β but to a lesser extent by IL-6 and TNFα. This is the first study to identify sema3C, sema3D and their receptors within the nucleus pulposus of intervertebral discs. Immunopositivity shows significant increases in semaphorin3C, 3D and their receptor neuropilin-2 in degenerate samples which were shown to contain nerves and blood vessels, compared to non-degenerate samples without nerves and blood vessels. Therefore data presented here suggests that semaphorin3C may have a role in promoting innervation and vascularisation during degeneration, which may go on to cause low back pain.

## INTRODUCTION

The intervertebral disc (IVD) is suggested to be the largest aneural and avascular tissue within the human body and is composed of three main anatomical regions; the gelatinous nucleus pulposus (NP) which is contained by the annulus fibrosus (AF) and the cartilaginous end plate (CEP). Chronic low back pain (LBP) is known to affect 80% of the population during their lifetime, and 40% of these cases are attributed to IVD degeneration [1]. Disc degeneration is characterised by the early loss of extracellular matrix (ECM) as well as altered biomechanical properties caused by an imbalance between anabolic and catabolic factors [2– 4]. Cytokines, in particular, interleukin-1 (IL-1) and tumour necrosis factor alpha (TNFα) are well known for their role within IVD degeneration [5–12], consequently leading to increased production of matrix degrading enzymes such as matrix metalloproteinases (MMPs); MMP-3 and -13, and a disintergrin and metalloproteinase with thrombospondin motifs (ADAMTS), -1, -4 and -5 [12–18]. The IVD is known to become increasingly vascularised by small blood vessels and innervated by peptide containing sensory nerve fibres, which are thought to elicit pain [19–21]. This being said, the mechanisms by which these processes occur are yet to be fully elucidated.

Current literature surrounding the mechanisms of low back pain (LBP) associated with neural ingrowth have focussed on neurotrophic factors which are known to promote the ingrowth and survival of sensory nerves, including nerve growth factor (NGF) and brain derived neurotrophic factor (BDNF) [19, 22–29]. To date however, limited studies have investigated the potential role of axonal guidance molecules known as the Semaphorins. There are 8 classes of semaphorins, yet class 3 Semaphorins are the only secreted members of the semaphorin family found within vertebrates [30]. Initially semaphorins were thought to function as repulsive guidance cues, yet Sema3B and Sema3C have been postulated to have bi-functional properties involved in promotion and repulsion of axons [31–33]. Class 3 semaphorins signal their response through two prominent semaphorin receptors; the neuropilins (NRP) and the plexins [34–38]. Whilst the majority of membrane bound semaphorins signal via plexins alone, class 3 semaphorins require the NRP as an obligate co-receptor, generating a high affinity holoreceptor complex [34, 37, 38].

Sema3A has previously been identified within human IVD samples by immunohistochemistry (IHC) [39]. Tolofari *et al*., (2010) identified immunopositive disc cells at the periphery of the outer annulus fibrosus (OAF) within non-degenerate discs, however the number of immunopositive cells decreased in the OAF and increased within NP cells in degenerate discs [39]. Suggesting that Sema3A is a natural inhibitor present within the non-degenerate disc, preventing innervation and vascularisation. Yet during degeneration, the loss of Sema3A at the periphery could potentially allow the inappropriate entry of nerves and blood vessels into the AF [39]. However to date no studies have investigated expression of the remaining members of the semaphorins or their receptors and whether this expression is associated with the presence of nerves and blood vessels. Hence this study aimed to identify class 3 semaphorins (Sema3A-F) and their receptors within human NP tissue and identify whether their expression was regulated by pro-inflammatory cytokines. Importantly this study aimed to identify whether their expression was associated with the presence of nerves and blood vessels.

## RESULTS

### Identification of class 3 semaphorins and their receptors within native NP tissue

**Figure 1:**
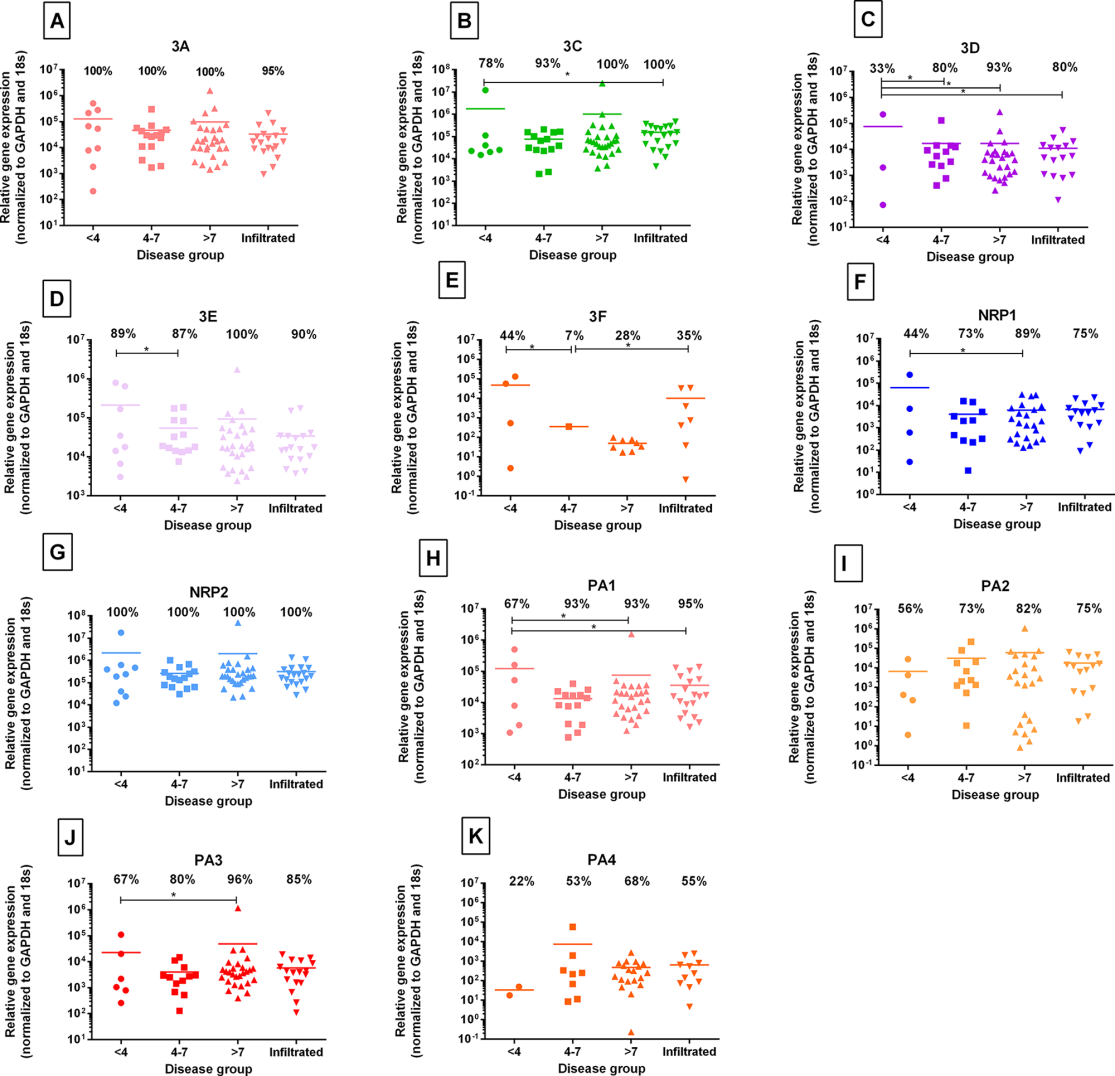
Identification of class 3 semaphorins and their receptors within native IVD tissue Percentage expression is represented as the number of disc samples in each cohort that expressed the target gene, with the mean value shown by the bar. Sema3A **A.** was present in 100% of non-degenerate and degenerate samples with 95% of infiltrated samples expressing Sema3A. Sema3C **B.** was present in 78% of samples graded < 4, however expression was present in 100% of severely degenerate (grade > 7) and infiltrated samples. Sema3D **C.** was expressed in 80% of moderately degenerate samples (grade 4–7) and 93% of severely degenerate samples (grade > 7). Sema3E **D.** was present in 89% of non-degenerate samples, increasing to 100% of samples in the severely degenerate cohort expressing Sema3E. Sema3F **E.** expression was present in 44% of non-degenerate samples, yet sema3F was only present in 1 sample (7%) of moderately degenerate samples. NRP1 **F.** expression levels increased from 44% expression in non-degenerate samples to 89% of severely degenerate samples expressing the receptor. NRP2 **G.** was present in 100% of samples regardless of disease classification. PlexinA1 **H.** was present in more degenerate samples than those graded as non-degenerate. PlexinA2 **I.** was present in 56% of non-degenerate samples, rising to 82% expression in severely degenerate samples. PlexinA3 **J.** was present in 96% of severely degenerate samples, and PlexinA4 **K.** was expressed by 22% of non-degenerate samples; increasing to 68% expression in the severely degenerate cohort. No significance was identified between expression levels and grades of degeneration. Statistical analysis was performed on proportions expressed *P = < 0.05*.

Directly extracted NP cells were investigated by QRT-PCR for the native expression of members of the class 3 semaphorins and their receptors, of which Sema3A, 3C, 3D, 3E and 3F along with NRP's and Plexins were identified within IVD tissue, irrespective of their classification. Sema3B was not identified in any sample. Sema3A was present in 100% of non-degenerate and degenerate samples, and 95% of infiltrated samples (Figure 1A). Sema3C and Sema3D were present in a greater number of degenerate samples compared to non-degenerate samples (*P* = < 0.05) (Figure 1B & 1C). Sema3C was present 78% of non-degenerate samples, rising significantly to 100% of infiltrated samples expressing sema3C (*P* = 0.0443) (Figure 1B). The proportion of samples expressing sema3D was significantly higher in the moderately degenerate (*P* = 0.0195), severely degenerate (*P* = 0.0004) and infiltrated (*P* = 0.0169) cohorts in comparison to non-degenerate cohort (Figure 1C). Sema3E was present in 100% of severely degenerate samples, a significantly higher proportion compared to expression in the moderately degenerate cohort (87%) (*P* = 0.05) (Figure 1D). Sema3F was expressed at low levels and present in 44% of non-degenerate samples, falling significantly to only 7% of moderately degenerate samples expressing sema3F (*P* = 0.0252) (Figure 1E). The proportion of severely degenerate samples expressing NRP1 was significantly higher than the number of non-degenerate samples expressing NRP1 (*P* = 0.0062) (Figure 1F). NRP2 was constitutively expressed by 100% of samples investigated, irrespective of disease classification (Figure 1G). PA1 was expressed by a significantly higher proportion of samples in the severely degenerate (*P* = 0.0448) and infiltrated cohorts (*P* = 0.0407) compared to non-degenerate samples (Figure 1H). PA2 (Figure 1I) and PA4 (Figure 1K) were expressed by a higher proportion of severely degenerate samples, compared to non-degenerate samples, yet this failed to reach significance. PA3 (Figure 1J) was expressed by 96% of severely degenerate samples, significantly higher than the number of non-degenerate samples expressing PA3 (*P* = 0.0197) (Figure 1J).

### Cytokine regulation of class 3 semaphorins within human NP cells, HDMEC and SH-SY5Y cells

**Figure 2:**
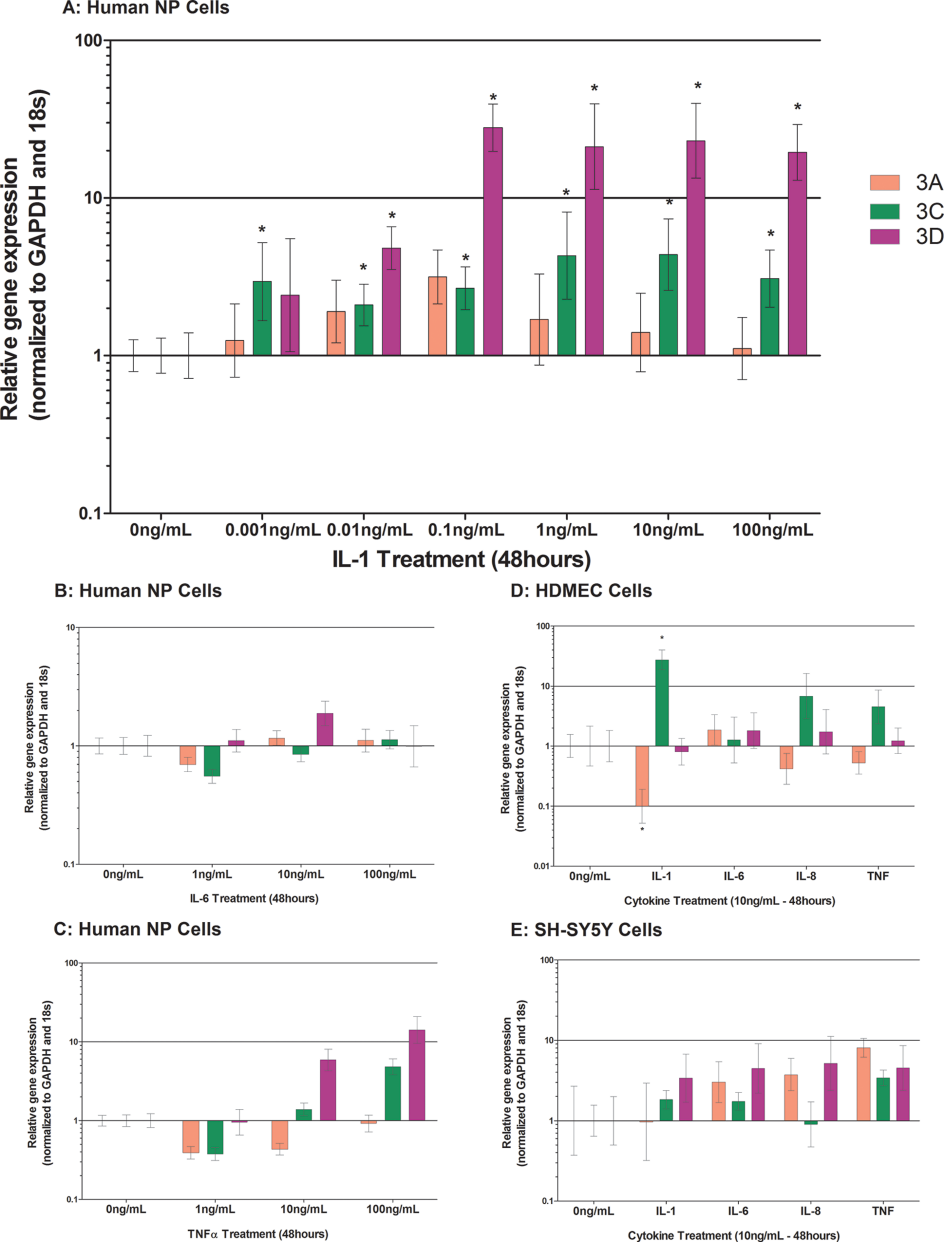
Cytokine regulation of Class 3 Semaphorins in human NP cells treated with IL-1β A. IL-6 B. and TNFα C. HDMECs D. and SH-SY5Y E. cells were treated with 10 ng/mL of IL-1β, IL-6, IL-8 or TNFα Sema3C and Sema3D were significantly up regulated by IL-1β treatment (*P* = ≤ 0.05) (A). IL-6 failed to regulate cytokine expression within human NP cells (B). TNFα caused a non-significant increase in Sema3C and Sema3D (C) IL-1β significantly up regulated Sema3C from HDMEC cells, and caused a significant down regulation in Sema3A (D). SH-SY5Y cell stimulation with all cytokines caused non-significant increases in Sema3A, Sema3C and Sema3D (E).

Following the identification of class 3 semaphorins and their receptors within native NP tissue, NP cells derived from degenerate IVDs were cultured in alginate and stimulated with pro-inflammatory cytokines, to determine modulation by inflammatory cytokines. Following IL-1β treatment for 48 hours, human NP cells significantly up regulated their expression of Sema3C at concentrations ≥ 1 pg/mL (*P* ≤ 0.05) (Figure 2A) and Sema3D at concentrations ≥ 100 pg/mL (*P* ≤ 0.0001) (Figure 2A). IL-6 and TNFα treatment in human NP cells failed to regulate semaphorins (*P* = > 0.05) (Figure 2B & 2C).

HDMEC stimulation with 10 ng/mL IL-1β significantly increased Sema3C (*P* = 0.0007) whilst sema3A was significantly decreased (*P* = 0.0071) (Figure 2D). IL-6, IL-8 and TNFα failed to regulate semaphorin expression in HDMEC or SHSY5Y cells, IL-1β also had no effect on SHSY5Y cells (Figure 2D & 2E).

### Cytokine regulation of neuropilins and plexins within human NP cells, HDMEC and SH-SY5Y cells

**Figure 3:**
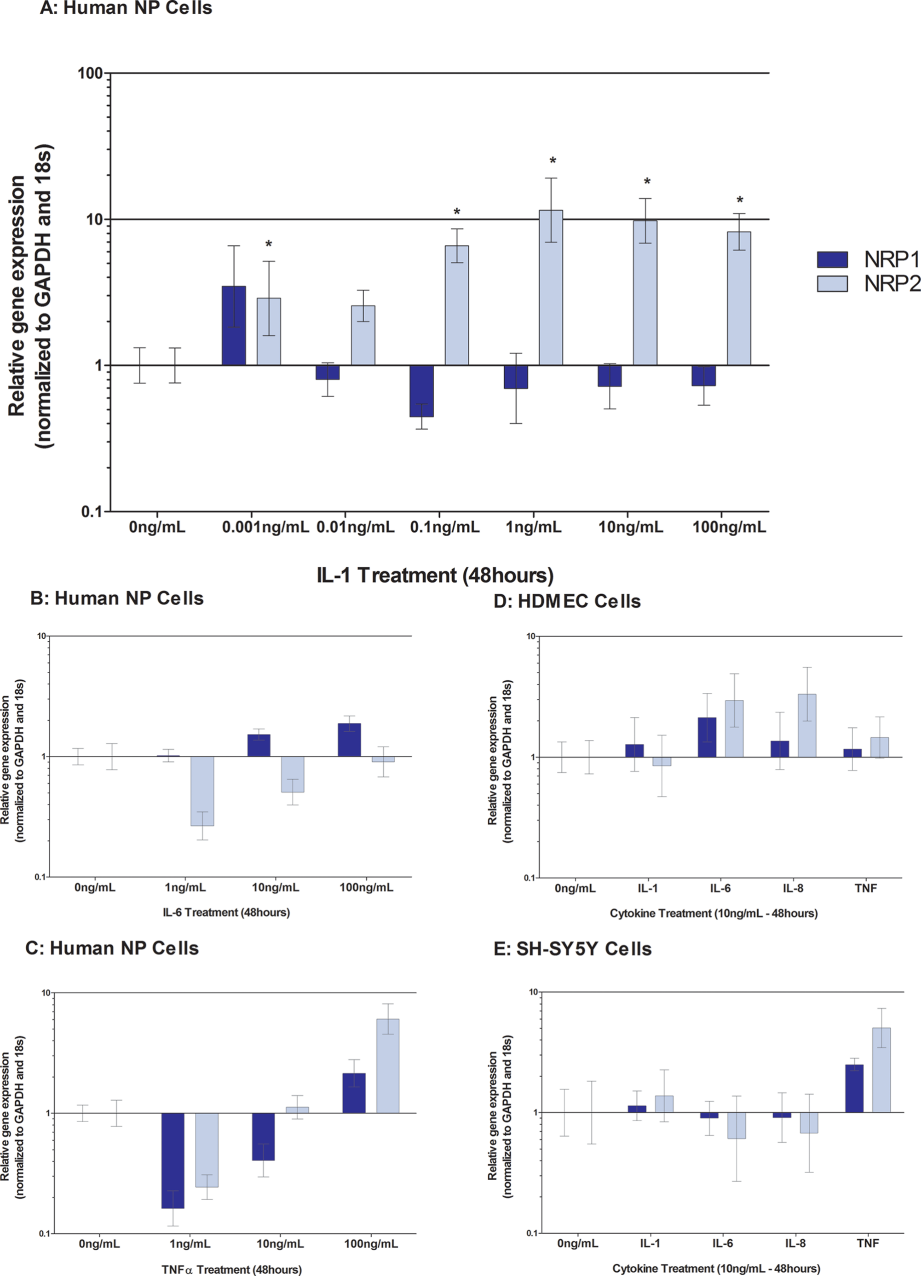
Cytokine regulation of Neuropilin-1 and Neuropilin-2 in human NP cells treated with IL-1 A. IL-6 B. TNFα C. and HDMECs D. and SH-SY5Y E Cells were treated with 10 ng/mL of IL-1, IL-6, IL-8 and TNFα. In human NP cells, IL-1β caused down regulation of NRP1, and a significant up regulation of NRP2 (*P* = ≤ 0.05) (A) Small but non-significant increases in NRP2 were seen in HDMECs in response to IL6 and IL-8 (D). TNFα stimulation appeared to cause a small increase in NRP1 and NRP2 expression, yet this was not significant (E)

IL-1β induced a significant up regulation of NRP2 in NP cells (*P* = ≤ 0.0001) (Figure 3A) however NRP1 was not significantly regulated and no regulation was observed within HDMEC or SHSY5Y cells (Figure 3D, 3E). Whilst IL-6, IL-8 and TNFα failed to significantly regulate NRP1 or NRP2 in all cell types investigated (Figure 3B–3E).

**Figure 4:**
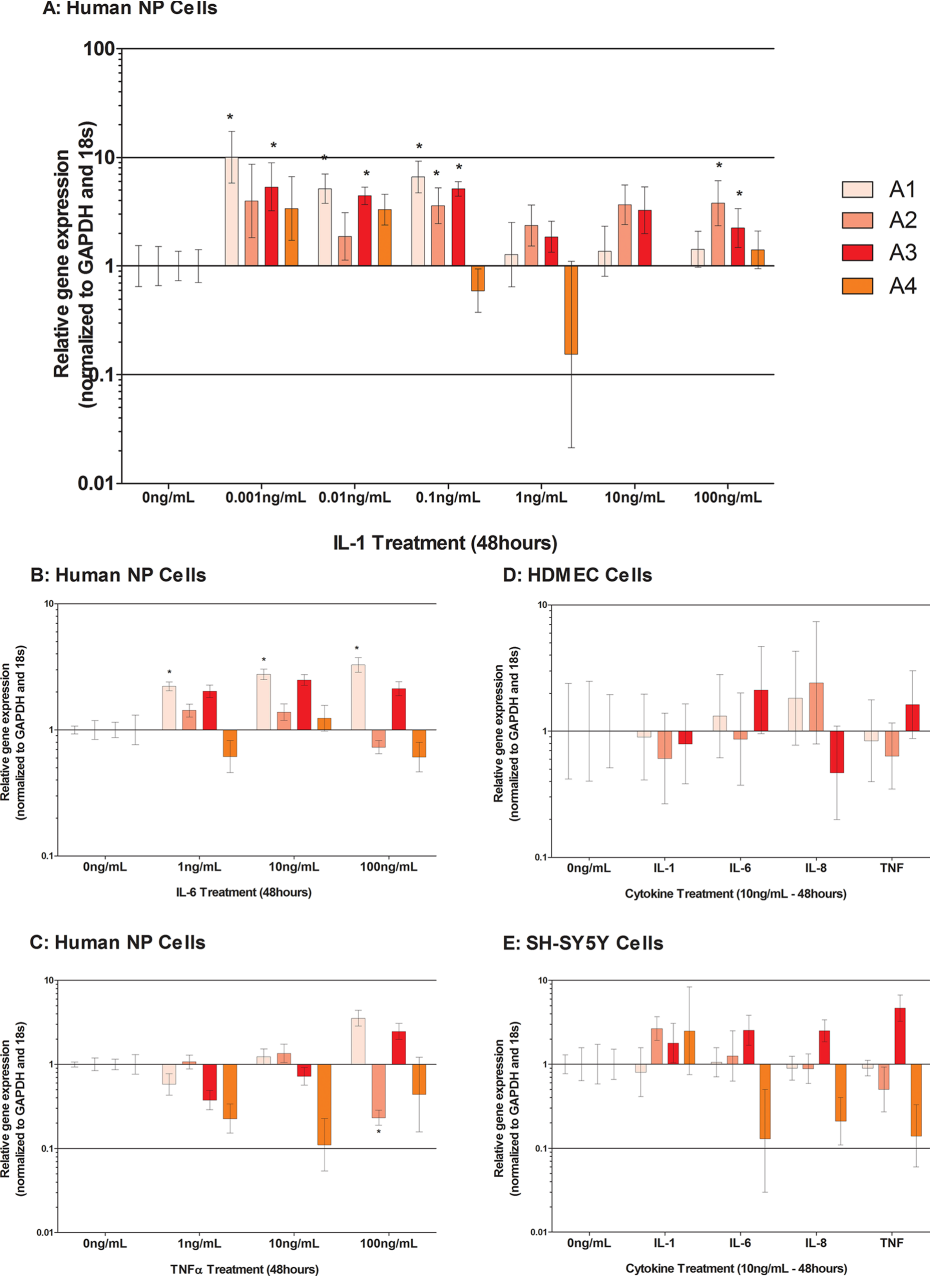
Cytokine regulation of Plexins (A1-A4) in human NP cells treated with IL-1 A. IL-6 B. TNFα C. and HDMECs D. and SH-SY5Y E Cells were treated with 10 ng/mL of IL-1, IL-6, IL-8 and TNFα. IL-1β (A) and IL-6 (B) significantly up regulated PA1 in human NP cells, whilst IL-1β also significantly up regulated PA2 and PA3 (A) in human NP cells. A non-significant decrease in PA4 was seen in response to IL-1β treatment at 1 ng/mL (A) PA2 was significantly down regulated in response to 10 ng/mL TNFα treatment in human NP cells (*P* = ≤ 0.05) (C) Plexin expression was not regulated by cytokines in HDMEC cells (D) Small but non-significant increases in PA3 expression were seen in SHSY5Y cells in response to all cytokines; however PA4 was non-significantly decreased by IL-6, IL-8 and TNFα (E)

All plexins were identified within native IVD tissue (Figure 1F–1I). Within human NP cells, PA1 PA2 and PA3 were significantly increased by IL-1β treatment (*P* < 0.05) (Figure 4A) whilst PA4 was not regulated (Figure 4A). IL-6 treatment significantly increased PA1 (*P* = 0.03) but failed to regulate other plexins (Figure 4B). TNFα induced a significant decrease in PA2 following 100 ng/ml stimulation (*P* = 0.0065), but failed to regulate other plexins (Figure 4C). Plexin expression was not regulated by cytokines within HDMEC cells (Figure 4D) or SH-SY5Y cells (Figure 4E).

### Immunodetection of semaphorins and their receptors within human IVD tissue

**Figure 5:**
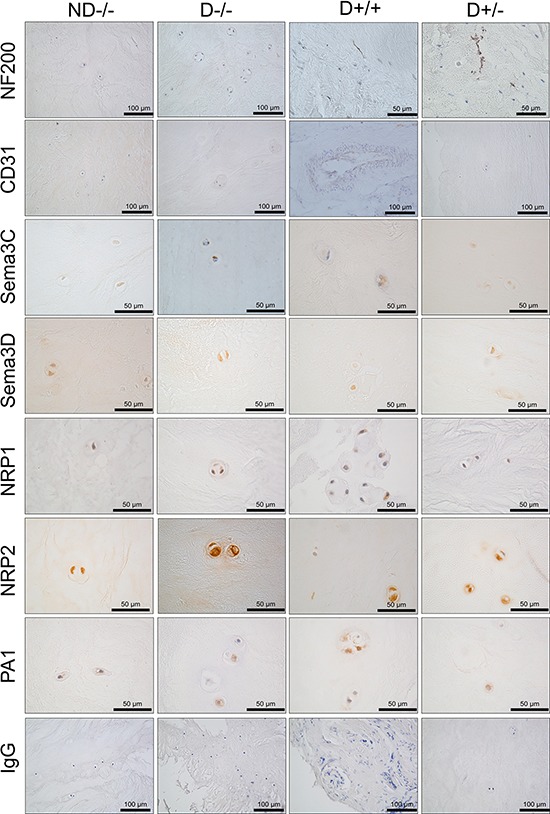
Immunopositivity of class 3 semaphorins and their receptors within the IVD Four groups were investigated for the expression of sema3C, sema3D and their receptors, NRP1, NRP2 and PA1 in tissues which were of differing grade of degeneration and whether nerve and/or blood vessels were present. (ND−/−: non-degenerate NF200−/CD31−. D−/−: Degenerate NF200−/CD31−. D+/+: Degenerate NF200+/CD31+. D+/−: Degenerate NF200+/CD31−).

Immunopositivity was first identified for NF200 and CD31 to determine patient samples with the presence of nerves (NF200+) and blood vessels (CD31+) within NP tissue (Figure 5A–5B). Semaphorin 3C, sema3D and their receptors NRP1, NRP2 and PA1 were investigated for their presence in NP tissue and their association with nerve and blood vessels (Figure 5C–5G). (Four groups were investigated: ND−/−: non-degenerate NF200−/CD31−. D−/− : Degenerate NF200−/CD31−. D+/+: Degenerate NF200+/CD31+. D+/−: Degenerate NF200+/CD31−), note specific grade of degeneration within each group was not seperated for investigation as this would have resulted in 7 groups and reduced the number of samples within each group.

**Figure 6:**
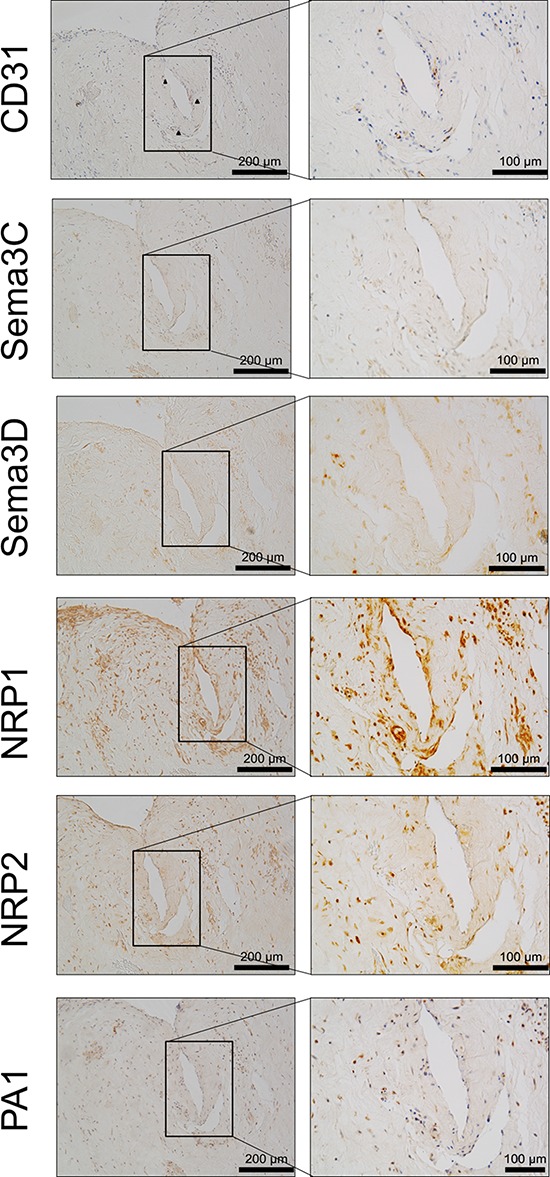
Co-localisation of CD31 and sema3C, sema3D and receptors NRP1, NRP2 and PA1 immunopositivity within human IVD tissue This demonstrates a link between localisation of CD31 and Sema3C, Sema3D, PA1 and the NRP's which are known to be co-receptors for VEGFR.

Co-localisation of CD31 and target molecules was performed on serial sections and demonstrates a strong co-localisation of CD31 and NRP1, NRP2 and PA1 (Figure 6). Sema3C and sema3D expression is not directly co-localised with the blood vessels, yet surrounding cells do express sema3C and sema3D (Figure 6). Nerve identification on tissues not stained with NF200 was difficult to localise, and thus co-localisation was not possible.

**Figure 7:**
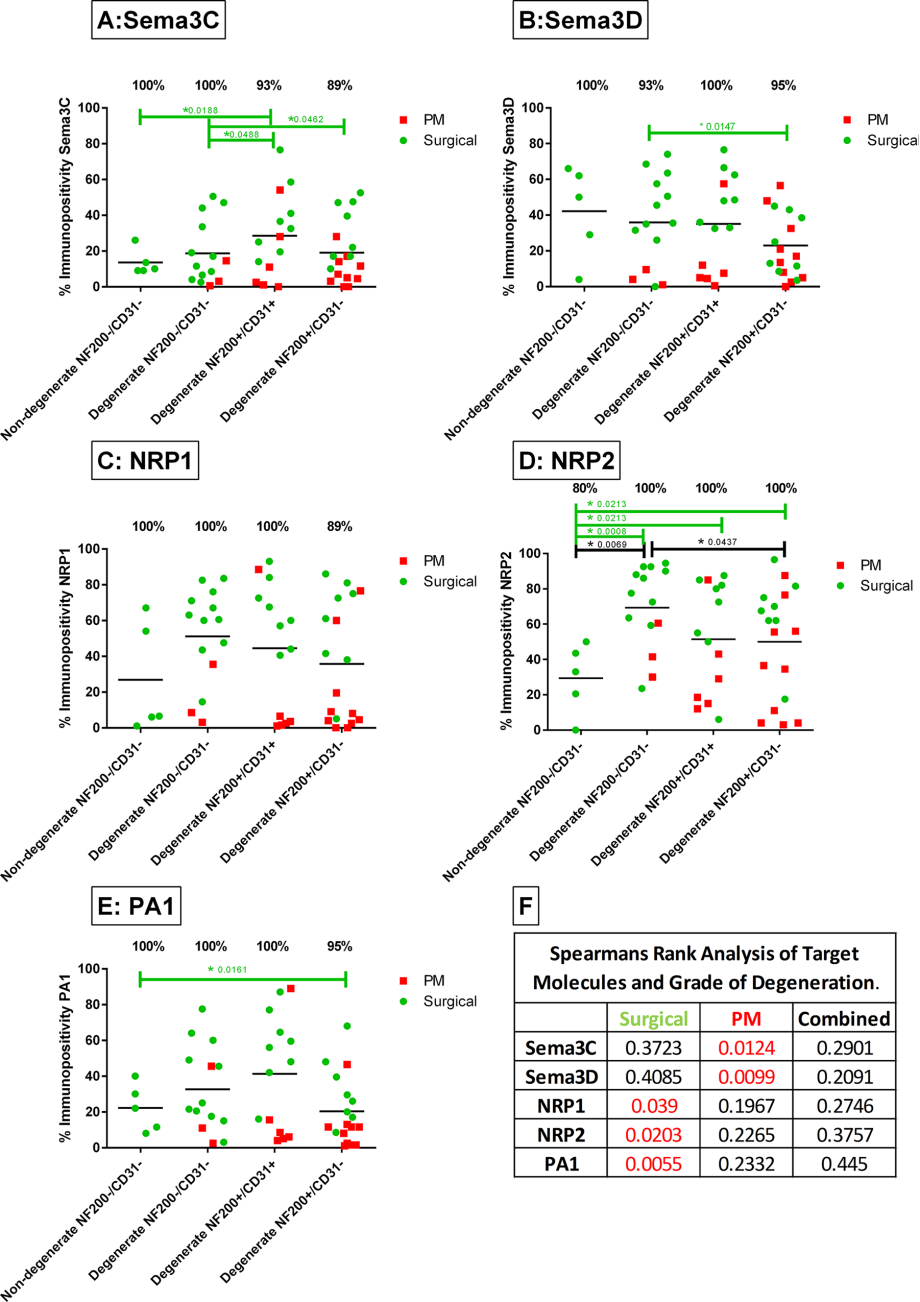
Immunopositivity of sema3C A. sema3D B. NRP1 C. NRP2 D. and PA1 E. in human NP tissues from surgically painful IVD's (green) and non-painful PM IVD's (red) Samples were split into different cohorts firstly based on histological degeneration and then on the identification of NF200+ and CD31+ tissue which indicates the presence of nerve and blood vessels. Immunopositivity for all molecules was generally higher in the surgically painful IVDs than in the PM samples, with significance reported in immunopositivity levels between the ND−/− and D+/+ for sema3C (A) and NRP2 (D) Correlation of immunopositivity and grade of degeneration was analysed and *P* values were recorded **F.** Sema3C and Sema3D immunopositivity showed a strong correlation with grade of degeneration in the PM samples alone, when the data was combined for surgical and PM discs, correlation was lost due to variation (F) NRP1, NRP2 and PA1 demonstrated strong correlation between immunopositivity and grade of degeneration in the painfully surgical samples, yet correlation was lost when data was combined with PM samples (F).

Expression levels of sema3C were significantly higher in the D+/+ group compared to the ND−/− group (*P* = 0.0118) (Figure 7A). PM samples demonstrate lower levels of expression of sema3C compared to surgically painful samples, particularly in the D−/− cohort (Figure 7A). A significant positive correlation between immunopositivity and grade of degeneration was seen in the PM cohort alone (*P* = 0.0124) (Supplementary Figure 1), yet when the data is combined with the surgical cohort, significance was lost due to variation seen in the surgical cohort (*P* = 0.2901).

Sema3D was expressed in 100% of ND−/− samples and D+/+ samples, decreasing to 93% of patients in the D−/− cohort (Figure 7B). Sema3D expression levels was significantly higher in the D−/− compared to D+/− group (*P* = 0.0147). Again, as with sema3C, sema3D expression levels in the PM samples in the D−/− and D+/+ were very low compared to the surgical painful samples, yet no significance was seen due to patient variation (Figure 7B). A significant correlation between sema3D expression and grade of degeneration was seen within the PM cohort alone (*P* = 0.0099) (Figure 7F).

NRP1 immunopositivity was detected in 100% of ND−/−, D−/− and D+/+ patients samples, yet in the degenerate cohort with nerves and lacking blood vessels, NRP1 expression levels decreased to 89% (Figure 7C). Expression levels between the cohorts did not show significant differences due to variation between the surgical and PM, as shown in red, the PM samples showed much lower levels of expression in each cohort (Figure 7C). Immunopositivity of NRP1 and grade of degeneration were significantly correlated in the surgical cohort (*P* = 0.039), yet no significance were seen within the PM cohort or when the samples were combined (Figure 7F).

NRP2 immunopositivity in the surgical cohort alone, showed significantly higher expression levels in the D−/− (*P* = 0.0008), D+/+ (*P* = 0.0213) and D+/− (*P* = 0.0213) cohorts compared to the ND−/− cohort. When surgical and PM samples were combined, expression levels in the D−/− group were significantly higher than in the ND−/− (*P* = 0.0069) group. Immunopositivity in the D−/− group was significantly lower than in the D+/− group (*P* = 0.0437) (Figure 7D). A strong positive correlation was shown between NRP2 immunopositivity and grade of degeneration in the surgical cohort alone (*P* = 0.0203), yet when the data was combined with the PM samples, correlation was lost (Figure 7F).

PA1 was expressed in 100% of samples in the ND−/−, D−/− and D+/+ and 95% D+/− samples (Figure 7E). Immunopositivity levels were significantly higher in the D+/− group compared to the ND−/− (*P* = 0.0161). PM samples demonstrated much lower levels of PA1 expression as opposed to the painful surgical samples (Figure 7E). PA1 expression levels show a significant positive correlation between immunopositivity and grade of degeneration in the surgical cohort (*P* = 0.0055), yet due to the lack of correlation in the PM cohort, significance was lost when the data was combined (Figure 7F).

### Correlations between semaphorin and their receptor expression in human NP cells

Positive correlations were evident when data from PM and surgical were combined for the expression of sema3C and their receptors; NRP1 (*P* = < 0.0001) (Supplementary Figure 1A), NRP2 (*P* = 0.0008) (Supplementary Figure 1B), and PA1 (*P* = < 0.0001) (Supplementary Figure 1C). Likewise, correlations between sema3D and its receptors were seen NRP1 (*P* = 0.0002) (Supplementary Figure 1D) NRP2 (*P* = 0.0003) (Supplementary Figure 1E) and PA1 (*P* = 0.0001) (Supplementary Figure 1F).

Positive correlation was identified between immunopositivity of sema3C and sema3D (*P* = < 0.0001) (Supplementary Figure 2A), NRP1 and NRP2 (*P* = < 0.0001) (Supplementary Figure 2B), NRP1 and PA1 (*P* = < 0.0001) (Supplementary Figure 2C), and NRP2 and PA1 (*P* = < 0.0001) (Supplementary Figure 2D).

## DISCUSSION

It is postulated that the ingrowth of nociceptive nerves and blood vessels into the degenerate IVD may be involved in the generation of chronic back pain [19, 28], yet the mechanisms involved in innervation and vascularisation of the IVD remain largely unknown. Recent studies have focused heavily on the presence of neurotrophic and angiogenic factors, in the hope of identifying a key player involved in the ingrowth into diseased discs [24, 25, 29, 43–46]. However only one paper to date has investigated the role of semaphorins which are thought to be inhibitory by nature to nerve and blood vessel ingrowth within the degenerate IVD [39]. Here, all class 3 semaphorins and their receptors at mRNA level were investigated, together with their regulation by cytokines known to be up regulated within the degenerate IVD. Further to this, sema3C, sema3D and their receptors were investigated for their protein expression within IVD tissues in association with nerve and blood vessels.

Members of the class 3 semaphorins are primarily known for their role in axonal pathfinding during development [47, 48], as well as having implications in angiogenesis and the immune system [49–51] amongst others [50].

Here, sema3A, sema3C, sema3D, sema3E, sema3F and their receptors the NRPs and PA1-A4 were identified at mRNA level in native NP cells. Although no significant changes in expression levels between the degenerate cohorts were identified, it is interesting that the majority of samples, particularly in the diseased state expressed these molecules. In contrast to the previous study, sema3A expression was evident in 100% of IVD tissues regardless of grade of degeneration, whereas Tolofari *et al*., (2010) showed sema3A expression in 57% of healthy NP and 33% degenerate NP samples [39]. In addition to this, NRP1 was expressed by 89% of our severely degenerate DE NP samples; yet Tolofari *et al*., (2010) did not see any expression of NRP1 in the healthy NP cohort but 60% of degenerate NP samples expressed NRP1. NRP2 was constitutively expressed by NP cells regardless of disease state in our study, however low levels were seen in both healthy and degenerate NP in the previous study. Plexin expression was evident in all disease states, yet Tolofari *et al*., (2010) did not identify PA1 in any sample, and only found PA2 in degenerate NP samples. The discrepancies in expression between the two studies could be due to the sensitivity of quantitative real-time PCR used here on more patient samples as opposed to standard real time PCR used in the previous study on 7 NP samples.

As the semaphorins are heavily involved in the repulsion of nerves and blood vessels in other systems and particularly during development, it was postulated that the expression of semaphorins would decrease in degenerate IVD's where innervation and angiogenesis is evident. Tolofari *et al.,* (2010) described increases in PGP9.5 (a nerve fibre marker) and CD31 mRNA expression which correlated with low sema3A mRNA expression in degenerate AF tissue, thus suggesting that sema3A is a natural inhibitor of nerve and blood vessel ingrowth in healthy AF [39], however increased expression was seen in the NP. Not only did we identify the presence of semaphorins at mRNA level, but furthered this with the native localisation and expression of sema3C and sema3D in association with nerve and blood vessels at protein level in the NP.

Immunohistochemistry analysis revealed strong positive correlations between sema3C and sema3D expression with grade of degeneration within the non-painful PM samples alone, interestingly however, correlation was lost when combined with the painful surgical samples due to varied expression. On the contrary, NRP1, NRP2 and PA1 demonstrated strong positive correlations with grade of degeneration in the surgically painful IVD's alone. This data goes against the original hypothesis that semaphorins would decrease as the severity of degeneration occurs. However this being said, highly degenerate IVD's may not necessarily be painful and/or contain nerves and blood vessels [4], which we have shown to be the case; hence here we investigated the expression in association with grade and the presence/absence of nerve and blood vessel ingrowth. Although all surgical samples used in this study were taken from patients experiencing pain, it proves difficult to determine the source of pain; it could be postulated that those tissues that were histologically graded as ND−/− or D−/− may be experiencing pain solely from compression or irritation of the nerve root due to herniation of the IVD. Whereas, samples that do have nerves and/or blood vessels present, may have a heightened response to pain due to the presence of nociceptive nerve fibres. Both sema3C and sema3D were identified in all NP samples in the ND−/− cohort, however the percentage immunopositivity for sema3C and sema3D increased in degenerate tissue samples with evidence of nerve and blood vessels. Surgically painful degenerate IVD's demonstrated a higher expression of both sema3C and sema3D compared to non-painful PM tissues; suggesting a possible attractive role rather than repulsion. Similarly, levels of NRP1 and PA1 from surgically painful degenerate IVD's were higher than those of non-painful PM tissue. NRP2 immunopositivity levels were more varied between non-painful PM samples. The higher immunopositivity levels in painful surgical samples indicate a more prominent role for semaphorins in painful surgical samples. It must be taken into account that the preparation of the surgical and PM samples were different, and the PM samples processed at Utrecht may have been subject to antigen destruction during decalcification which could explain the overall decreased levels of immunopositivity in these samples. Sema3C and sema3D immunopositivity demonstrated strong correlations with their receptors. This data indicates that sema3C and sema3D may have an active role in degeneration as there is a corresponding up regulation in their receptors. Sema3C has been described as a bi-functional semaphorin as it can also attract cells such as mouse glomerular endothelial cells, in a similar fashion to that seen by VEGF [52]. In this study, all samples where nerves and blood vessels were absent were positive for sema3C. Initially, this suggests that sema3C may have a protective role against nerve and blood vessel ingrowth. However, in the degenerate cohorts with evident nerve and blood vessel ingrowth, immunopositivity levels were significantly higher compared to negative/negative samples, indicating that sema3C may have a bi-functional role within the IVD to promote ingrowth of blood vessels and nerve fibres.

Although sema3D has not been characterised as well as other members of the class 3 semaphorins, it too has been shown to possess chemorepellent and chemoattractant properties towards neurons within the CNS of zebrafish depending on the composition of the NRP molecule [53]. Immunopositivity of sema3D was seen in very high proportions of IVD samples regardless of whether nerves and blood vessels were present. However levels of sema3D expression in the degenerate cohort with nerves but no blood vessels, was lower than in the degenerate negative/negative cohort indicating that sema3D may be involved in the chemoattraction of nerves but not blood vessels.

Sema3C and sema3D both bind to NRP1 and NRP2 with equal affinity, immunopositivity levels of NRP1 and NRP2 were higher in the degenerate negative/negative samples compared to the non-degenerate negative/negative samples suggesting a possible role for the NRPs in degeneration. NRP's are a co-receptor for VEGF as well as semaphorins, and VEGF is known to be up regulated by cytokines within human NP cells within the degenerate disc [29]. This study demonstrated a strong co-localisation with endothelial cells and NRP1, NRP2 and PA1 which are known co-receptors for VEGFR binding.

Regulatory studies performed here demonstrate for the first time that class 3 semaphorins and their receptors are susceptible to regulation by inflammatory cytokines particularly IL-1β, in human NP cells. Cytokines are well known for their role in human IVD degeneration, and they have recently been shown to regulate the expression of neurotrophic factors, particularly NGF and BDNF in human NP cells [22, 26, 29]. This suggests a function for these molecules in the promotion and survival of nerve and blood vessel ingrowth into the degenerate IVD. However, as indicated above there may be a role for the semaphorins in the advocacy of nerve and blood vessel ingrowth as their expression from human NP cells in degenerate tissue with/without nerves and blood vessels differs to non-degenerate negative/negative samples.

Interestingly, sema3C and sema3D were more responsive to cytokine stimulation than other more characterised semaphorins, showing significant up regulations in response to as little as 10 pg/mL IL-1β stimulation. Semaphorins were not regulated by IL-6 or TNFα in human NP cells, thus suggesting a key role for IL-1β in this process. Likewise, regulation of semaphorin receptors was more prominent in response to IL-1β stimulation than IL-6 or TNFα. Stimulation with IL-1β induced NRP2 and plexin expression. A previous study relating to human proximal tubular cells demonstrated similar trends after IL-1β stimulation in that NRP1 decreased, and NRP2 increased [54]. From the data presented here, it could be hypothesised that cytokines within the degenerate NP, stimulate the up regulation of sema3C and sema3D and, as previously shown NGF and VEGF [29] which are factors known to promote the survival and proliferation of nerve and blood vessels. Additionally IL-1β increased sema3C expression in endothelial cells which could intensify the chemoattraction of nerves along with the expression of NGF which we already know endothelial cells present in the human NP express [19]. We have shown that a nerve cell line (SHSY5Y) expresses NRP1, NRP2 and PA1-A4, however; although there is an up regulation of semaphorins from NP cells upon cytokine stimulation, the receptors are not regulated by cytokines in a nerve cell line or endothelial cells suggesting that nerve and blood vessels within the diseased IVD may not respond to cytokines from NP cells. The responses of NP, nerve and blood vessels to semaphorins need to be further investigated to identify if the receptors are active and able to exert the effects of semaphorins in the environment of the disc.

The interplay between biological and mechanical influences within the degenerative process has recently become a focus point within research, with many groups investigating the link between mechanical affects and biological outcomes. It is widely thought that multiple factors are involved in the generation of LBP, recent studies have highlighted a potential relationship between modic changes [55, 56] and low grade bacterial infection [57] in the production of pain, as both compromise the structural integrity of the vertebral body.

A possible initiating factor of IVD degeneration is the disruption of the vertebral end plate; this is known to result in increased catabolic enzyme production which degrade matrix molecules such as aggrecan. The loss of such proteoglycans is primarily known for its effect on disc hydration, yet the loss of disc height and pressure alterations within the NP can lead to extensive fissuring seen in degenerate IVD's [58]. Stefanakis *et al*., (2012) reported strong localisation of nerve and blood vessels to annular fissures due to its conducive environment to nerves and blood vessels [58]; hence providing a likely route into the deeper layers of the IVD. Data presented here show that nerves and blood vessels were present both next to and distant from fissures, yet it has to be taken into account that this study is based on 10 μm sections from herniated tissues, and PM samples. Ideally whole human IVDs would have been serially sectioned and stained to form a 3D model highlighting the pathway and localisation of nerve and blood vessels within the IVD. Recent studies by Gawri *et al.,* (2014) demonstrated an increased production of inflammatory factors such as NGF, TNFα and IL-6 from IVD cells under high mechanical strain [43]; thus suggesting that abnormal strain on the human IVD could potentiate or drive the inflammatory process that occurs during degeneration which may advocate the ingrowth of nerves and blood vessels. Results presented here, demonstrate an increased production of semaphorins and their receptors (also known to be involved in angiogenesis) in response to such inflammatory factors. This taken alongside recent studies focussing on mechanical cues could implicate a multifactorial process which ultimately accelerates disc degeneration.

## MATERIALS AND METHODS

### Human tissue

Human IVD tissue was obtained from surgery or post mortem examination (processed within 72 hours after death) with informed consent of the patient or relatives. Ethical approval was obtained from Sheffield Research Ethics Committee (09/H1308/70) for 89 surgical IVD samples from 89 individuals, and 4 post mortem (PM) samples from 2 individuals, and 18 PM samples from the department of Pathology/UMCU Biobank, UMC Utrecht, used in line with the code of proper secondary use of human tissue (Supplementary Table 1).

### Tissue processing

Tissue processed at Sheffield Hallam University consisting of AF and NP was fixed in 10% neutral buffered formalin, and processed to paraffin wax. Tissue processed at Utrecht was obtained 24 hours after death and IVD slices were decalcified in Kristensen's solution (50% formic acid, and 68 g/l sodium formate) in a microwave at 150 W and 50°C for 6 hours as previously published [40]. Sections were dehydrated and rinsed in xylene before being embedded to paraffin wax. Following embedding, 4 μm sections cut and histologically graded using previously published criteria [12, 29].

### Investigation of class 3 semaphorins and their receptors within native NP cells

Samples from degenerate IVD tissue were obtained from 69 patients undergoing microdiscectomy for the treatment of low back pain which mainly consisted of NP tissue, and 2 PM samples from 1 patient were used. NP tissue was separated from contaminating AF and CEP and digested with 2 U/ml protease (Sigma, UK) in DMEM for 30 minutes at 37°C, and washed twice with DMEM. Following this, NP cells were isolated using 2 mg/mL collagenase type 1 (Sigma, UK) for 4 hours at 37°C. Cells were passed through a 40 μM cell strainer (Invitrogen, UK) as previously published [41]. Following extraction cells were either used for direct RNA extraction or cell culture. RNA and cDNA was synthesised as previously published [42] [42]. Target genes were investigated using QRT-PCR.

Quantitative real-time polymerase chain reaction (QRT-PCR) was conducted on 9 non-degenerate samples (grade < 4) from 9 patients, 15 moderately degenerate samples (grade grade 4-7) from 15 patients, and 28 severely degenerate samples (grade > 7) from 28 patients, and 20 samples with evident infiltration from patients experiencing pain (Supplementary Table 1).

QRT-PCR was performed on a StepOnePlus™ Real-Time PCR System (Applied Biosystems, UK) in order to investigate gene expression levels of Semaphorin3A-F and their receptors, NRP1 and NRP2, along with PlexinA1-A4 (PA1-PA4) (Pre-designed primer/probe mixes Applied Biosystems, UK) within directly extracted samples from degenerate and non-degenerate samples. Glyceraldehyde-3-phosphate dehydrogenase (GAPDH) and 18S (Applied Biosystems, UK) were used as housekeeping genes to allow normalisation. Ten microliter reactions were prepared using TaqMan Universal PCR Master Mix (Applied Biosystems, UK). Results were analysed using the 2^−ΔCt^ method and presented as relative gene expression normalised to the average C_T_ for the two housekeeping genes.

### Cytokine regulation of class 3 semaphorins and their receptors in human NP cells, SH-SY5Y cells and HDMEC cells

#### NP cell culture

Following extraction NP cells were cultured for expansion in monolayer in DMEM supplemented with 10% v/v heat inactivated foetal calf serum (FCS), 100 U/ml Penicillin, 100 μg/ml Streptomycin, 250 ng/ml amphotericin, 2 mM glutamine (Invitrogen, U)K and 50 μg/ml ascorbic acid (Sigma Aldrich, UK) (Complete cell culture media) and maintained at 37°C in a humidified atmosphere containing 5% CO_2_. Complete culture media was changed every 3 days. Following expansion in monolayer up to passage 2, NP cells were resuspended in 1.2% medium viscosity sodium alginate (Sigma Aldrich, UK) at 4 × 10^6^ cells/ml and alginate beads as previously published [12]. All NP cells used in this experiment were cultured in alginate for 2 weeks prior to cytokine treatments to enable cells to regain native cellular phenotypes [12].

#### Monolayer SH-SY5Y and HDMEC cell culture

SH-SY5Y neuroblastoma cells were differentiated by a 7 day pre-treatment with 5 μM of retinoic acid prior to seeding into a 6 well plate at a cell density of 5 × 10^5^ cells/well. Human dermal microvascular endothelial cells (HDMECs) were seeded into 6 well plates at a density of 1 × 10^6^ cells/well in complete HDMEC growth factor media (PromoCell, UK).

### Cytokine treatments

Following 2 weeks culture in alginate beads, NP cells were treated with pro-inflammatory cytokines: IL-1β, IL-6 and TNFα (Peprotech, UK) for 48 hours. NP cells were treated with IL-6 and TNFα at 0, 1, 10 and100 ng/mL in serum free media in triplicate on 3 independent patients. IL-1β treatments were performed on 5 patients in triplicate (0, 1, 10, 100 ng/mL) and on 3 patients in triplicate at a wider concentration range (0, 0.001, 0.01, 0.1, 1, 10 and 100 ng/mL) for 48 hours to identify dose responses. SH-SY5Y and HDMEC cells were allowed to adhere for 24 hours prior to stimulation with 10 ng/mL IL-1β, IL-6, IL-8 or TNFα for 48 hours.

Following cytokine treatments, alginate beads were washed in 0.15 M NaCl and dissolved by application of alginate dissolving buffer (55 mM sodium citrate (Na_3_C_6_H_5_O_7_), 30 mM Na EDTA and 0.15 M NaCl at pH6.8) for 15 minutes at 37°C followed by digestion of extracellular matrix in 0.06% w/v collagenase type 1 (Sigma, UK) for 30 minutes. RNA was extracted and cDNA synthesised and QRT-PCR used to investigate expression levels of semaphorins and their receptors as described above.

### Immunohistochemistry

Initial investigations to identify NF200 (neurofilament 200, nerve marker) and CD31 (blood vessel marker) immunopositivity was performed in order to further investigate the relationship between the presence of nerve and blood vessels and the expression of factors involved in their survival in human NP tissue.

Tissues samples were selected which contained NP and AF tissue and were grouped into the following categories based on the identification of nerves (NF200+) and blood vessels (CD31+) in tissues which had been histologically graded. Non-degenerate tissues (grade < 4) NF200−/CD31− (ND−/−)*(n = 5)*, Degenerate tissues (grade > 4) NF200−/CD31− (D−/−)*(n = 14)*, Degenerate (grade > 4) NF200+/CD31+ (D+/+)*(n = 14)* and Degenerate (grade > 4) NF200+/CD31− (D+/−) *(n = 19)*. No disc samples were identified which displayed NF200−/CD31+ profile and hence this group was not investigated. Post mortem tissue was utilised to enable localisation of nerves and blood vessels within the different regions.

Studies were conducted on the above cohorts to investigate the expression of class 3 semaphorins, Sema3C and Sema3D and their receptors NRP1, NRP2 and PA1 and their association with tissues with nerves and blood vessels using immunohistochemistry as per Le Maitre *et al*., (2004) [13].

Briefly, four micron sections were dewaxed, rehydrated, and endogenous peroxidase blocked. Antigen retrieval methods were performed (Supplementary Table 2). Following TBS washes, nonspecific binding sites were blocked at room temperature for 2 hours with either 25% w/v goat or rabbit serum (Abcam, UK) in 1% w/v bovine serum albumin (Sigma, UK) in TBS (Supplementary Table 2). Sections were incubated overnight at 4°C with either mouse or rabbit polyclonal antibodies (Supplementary Table 2). Negative controls in which rabbit and mouse IgGs (Abcam, UK) replaced the primary antibody at an equal protein concentration were used. Slides were washed in TBS and a 1:500 dilution of 1% w/v BSA/TBS biotinylated secondary antibody was applied for 30 minutes at room temperature (Supplementary Table 2). Binding of the secondary antibody was disclosed with streptavidin-biotin complex (Vector Laboratories, UK) technique with 0.08% v/v hydrogen peroxide in 0.65 mg/mL 3, 3′-diaminobenzidine tetrahydrochloride (Sigma, UK) in TBS. Sections were counterstained with Mayer's haematoxylin (Leica, UK), dehydrated, cleared and mounted with Pertex (Leica, UK).

All slides were visualised using an Olympus BX60 microscope and images captured using a digital camera and software program QCapture Pro v8.0 (MediaCybernetics, UK). Evaluation of IHC staining was performed by counting a minimum of 200 NP cells as immunopositive or immunonegative; immunopositive cells were expressed as a percentage of total count.

### Statistical analysis

Data was shown to be non-parametric and hence a Kruskall-Wallis with Conover-Inman post hoc analysis test was used to identify significant differences between cytokine treatments on human NP cells as well as differences between patient cohorts investigated by IHC *(P ≤ 0.05)*. Two independent proportionality tests were performed to identify differences between the proportions of samples expressing the target molecules in the DE sample cohorts *(P ≤ 0.05)*. Linear regression analysis was performed to assess the correlation between target molecules used in IHC *(P ≤ 0.05)*.

## CONCLUSION

Previous research into painful intervertebral discs found associations with nerve and blood vessels and painful discs, with suggestions that blood vessels enter the IVD first, expressing NGF which promotes the in growth of nerves [19, 28]. Yet research presented here, suggests that nerves are able to innervate the NP without endothelial cell protrusion first; further research on a larger cohort of samples should be performed to confirm this finding. The current study suggests that members of the class 3 semaphorins may be implicated in the ingrowth of nerves and blood vessels in the degenerate NP of surgically painful IVDs, in particular sema3C and sema3D which according to previous research in other systems are thought to have potential chemoattractive functions. Further research is required to confirm the function of these molecules on nerves and blood vessels in conjunction with human NP cells, in a degenerate environment. To conclude, this study implicates a role for sema3C and sema3D in the innervation and vascularisation of the human IVD during degeneration, which is postulated to be a cause of chronic low back pain.

## SUPPLEMENTARY FIGURES AND TABLES



## Abbreviations

ADAMTs: A Disintigrin And Metalloproteinase with Thrombospondin motifs
AF: Annulus Fibrosus
BDNF: Brain Derived Neurotrophic Factor
CEP: Cartilaginous End Plate
H&E: Haematoxylin and Eosin
HDMEC: Human Dermal Microvascular Endothelial Cells
IL-1: Interleukin 1
IL-6: Interleukin 6
IL-8: Interleukin 8
IVD: Intervertebral Disc
LBP: Low Back Pain
MMP: Matrix Metalloproteinase
NGF: Nerve Growth Factor
NP: Nucleus Pulposus
NRP: Neuropilins
PA1: Plexin A1
PA2: Plexin A2
PA3: Plexin A3
PA4: Plexin A4
PM: Post Mortem
QRT-PCR: Quantitative Real Time Polymerase Chain Reaction
Sema: Semaphorins
TNF: Tumour Necrosis Factor
VEGF: Vascular Endothelial Growth Factor
