# Supplementary material for: Class 3 semaphorins expression and association with innervation and angiogenesis within the degenerate human intervertebral disc
Source: Oncotarget. 2015 Jun 15;6(21):18338–54. doi: 10.18632/oncotarget.4274 (PMC4621894; doi:10.18632/oncotarget.4274)
Supplement: Supplementary file 1 [file oncotarget-06-18338-s001.pdf]

SUPPLEMENTARY FIGURES AND TABLES

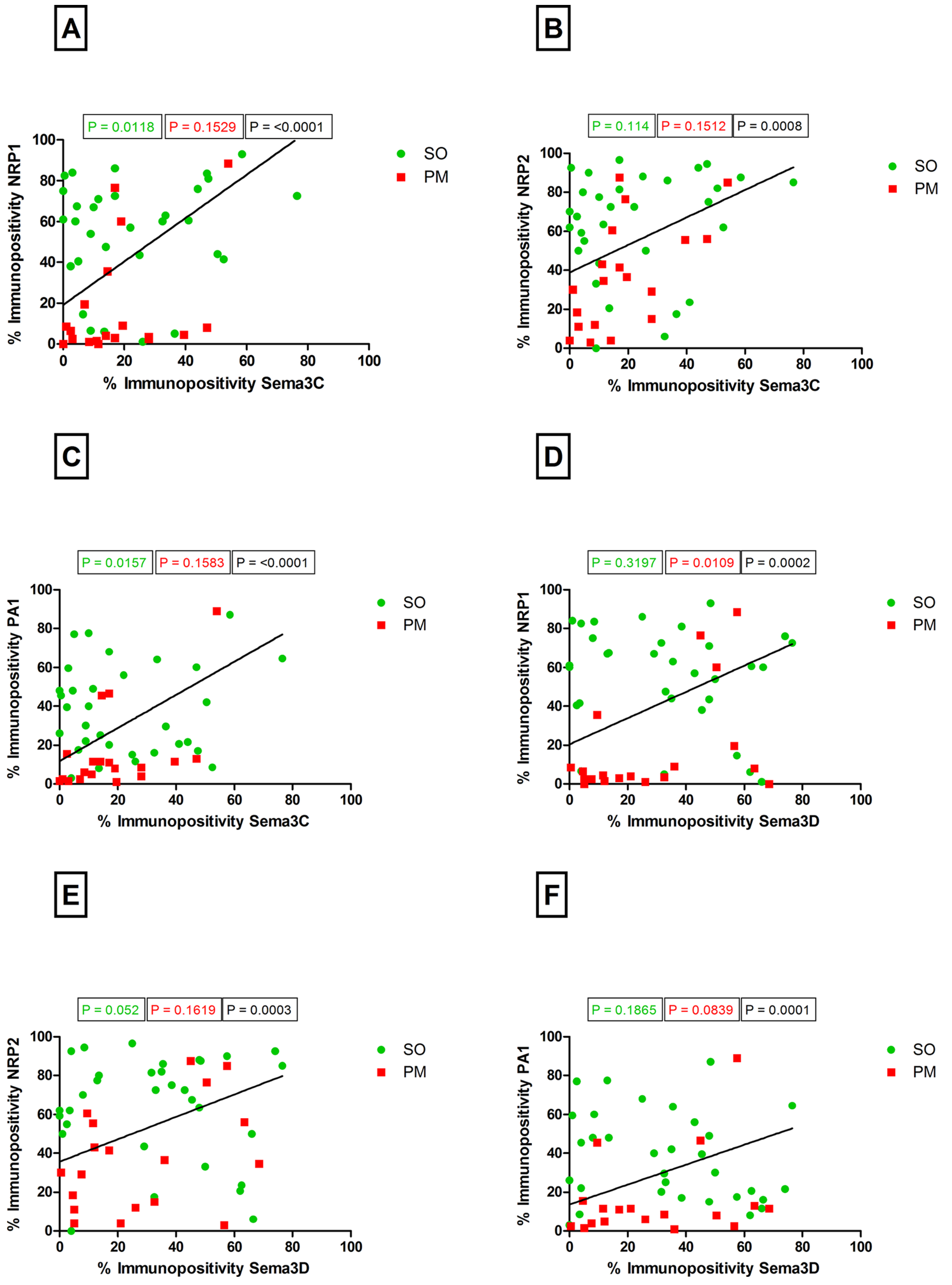

Supplementary Figure 1: Correlation of immunopositivity of sema3C against its receptors NRP1 A. NRP2 B. and PA1 C. demonstrated significant strong positive correlations in PM and surgical NP tissues. Strong positive correlations were evident between immunopositivity of sema3D against NRP1 **D.** NRP2 **E.** and PA1 **F.**

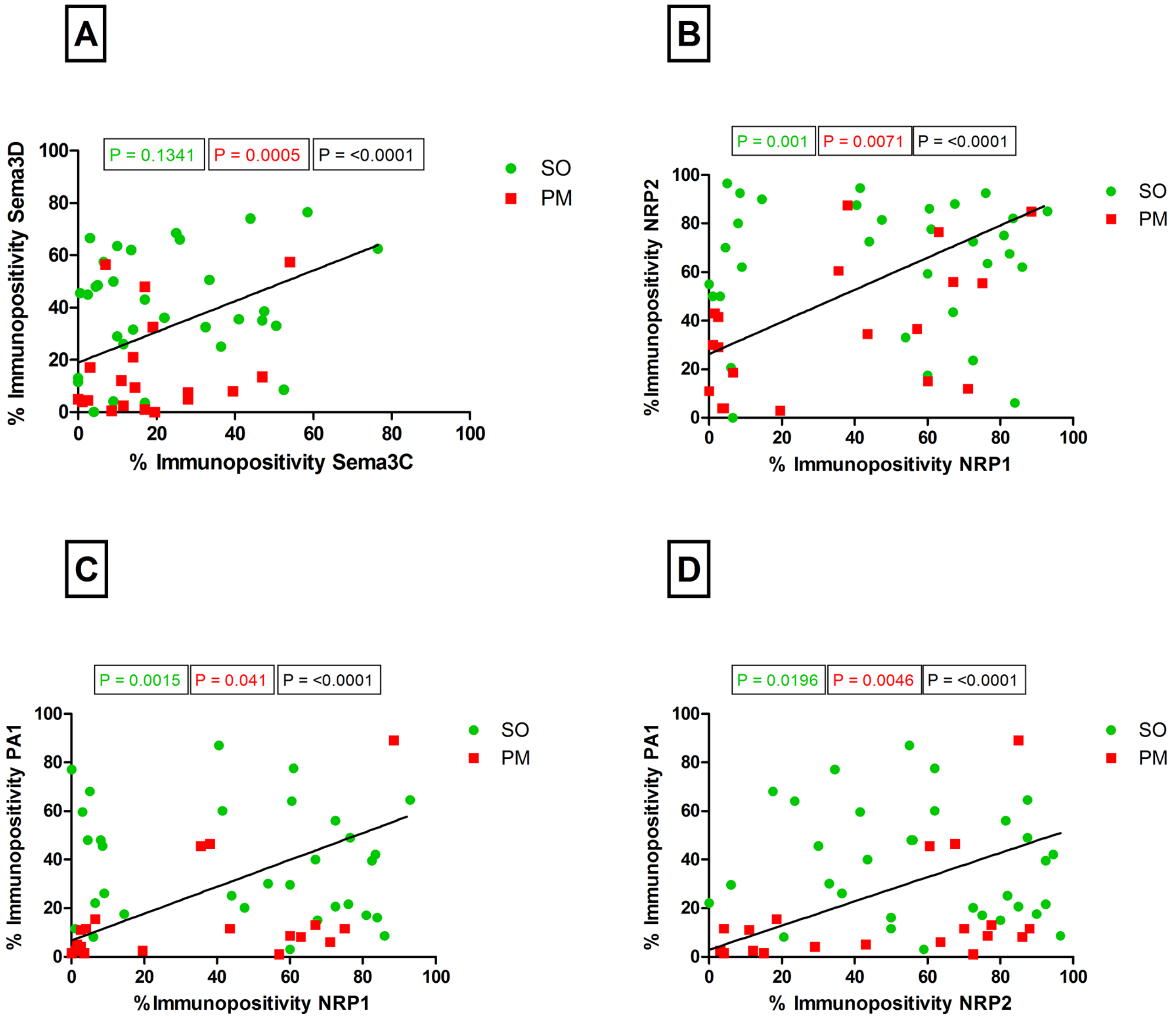

Supplementary Figure 2: Correlation of immunopositivity of sema3C against sema3D A. NRP1 against NRP2 B. NRP1 against PA1 C. and NRP2 against PA1 D. All correlations demonstrated significant strong positive correlations in PM and surgical NP tissues.

Supplementary Table 1: Patient details of human IVD samples used in this study. Samples marked PM (SHU) were processed at Sheffield Hallam University, and samples marked PM (UT) were processed in Utrecht. Non-degenerate (ND) grade < 4; Mildy-Degenerate (MD) grade 4–7; Severely-Degenerate (SD) grade > 7; Infiltrated cohort (I). Sample cohorts used for immunohistochemistry (IHC): ND-/- Non-degenerate, no nerves and no blood vessels; D-/- Degenerate no nerves, no blood vessels; D+/- Degenerate with nerves, no blood vessels; D+/+ Degenerate with nerves and blood vessels

Reference		Source	Age	IVD Level	IVD Intact?	Average Grade	Classification	DE	Cytokine Treatments	IHC	Cohort
**HD**	1	Surgical	42	L4/L5	No	2.5	I	✓		✓	ND -/-
**HD**	2	Surgical	40	L5/S1	Yes	7.0	I	✓			
**HD**	3	Surgical	25	L4/L5	Yes	4.0	MD	✓			
**HD**	5	Surgical	33	L5/S1	Yes	8.5	SD	✓		✓	D-/-
**HD**	9	Surgical	32	L5/S1	Yes	2.0	ND	✓		✓	D-/-
**HD**	17	Surgical	45	L5/S1	Yes	4.0	ND	✓			
**HD**	21	Surgical	26	L4/L5	Yes	4.0	I	✓			
**HD**	25	Surgical	20	L4/L5	No	3.0	I	✓			
**HD**	26	Surgical	40	L5/S1	No	5.0	I	✓			
**HD**	30	PM	45	L4/L5	Yes	2.0	ND	✓			
**HD**	31	PM	45	L3/L4	Yes	3.0	ND	✓			
**HD**	33	Surgical	48	L4/L5	No	10.0	SD	✓			
**HD**	34	Surgical	26	L5/S1	No	11.0	SD	✓			
**HD**	36	Surgical	33	L5/S1	Yes	9.0	I	✓			
**HD**	38	PM (SHU)	74		Yes	7.5				✓	D+/-
**HD**	39	PM (SHU)	74		Yes	11.5				✓	D+/+
**HD**	44	Surgical	42	L5/S1	Yes	3.0	ND	✓			
**HD**	45	Surgical	36	L5/S1	Yes	8.0	SD	✓			
**HD**	53	Surgical	38	L5/S1	No	7.0	MD	✓		✓	D+/+
**HD**	54	Surgical	28	L4/L5	Yes	9.0	SD	✓	✓		
**HD**	56	Surgical	43	L5/S1	No	7.0	MD	✓			
**HD**	57	Surgical	44	L5/S1	Yes	7.0	MD	✓			
**HD**	58	Surgical	28	L5/S1	No	8.0	SD	✓			
**HD**	59	Surgical	35	L5/S1	Yes	8.0	SD	✓			
**HD**	60	Surgical				5.0	MD			✓	D+/+
**HD**	61	Surgical	43	L5/S1	No	4.0	MD	✓			
**HD**	63	Surgical	42	L5/S1	No	9.0	SD	✓		✓	D-/-
**HD**	65	Surgical	43	L4/L5	No	10.0	SD	✓			
**HD**	66	Surgical	62	L3/L4	Yes	9.0	I	✓		✓	D-/-
**HD**	67	Surgical	39	L4/L5	Yes	5.0	MD			✓	D-/-

(*Continued*)

Reference		Source	Age	IVD Level	IVD Intact?	Average Grade	Classification	DE	Cytokine Treatments	IHC	Cohort
**HD**	74	Surgical		L4/L5	No	7.5	SD			✓	D-/-
**HD**	75	Surgical	40	L3/L4	Yes	7.0	MD	✓			
**HD**	78	Surgical	55	C3/C4	Yes	10.0	SD			✓	D-/-
**HD**	79	Surgical	45	L4/L5	No	3.0	ND	✓	✓		
**HD**	84	Surgical	38	C6/C7	Yes	5.0	MD			✓	D+/-
**HD**	85	Surgical	49	L2/L3	No	7.0	I	✓		✓	D+/+
**HD**	86	Surgical	40	L5/S1	No	3.0	I	✓			
**HD**	89	Surgical	21	L5/S1	No	4	MD	✓	✓	✓	
**HD**	90	Surgical				11.0	SD			✓	
**HD**	92	Surgical	38	L5/S1		3.5	ND	✓		✓	D+/-
**HD**	93	Surgical	38	L5/S1		7.0	MD	✓			
**HD**	94	Surgical	66	L5/S1	Yes	6.0	MD	✓			
**HD**	95	Surgical	39	L5/S1	Yes	6.0	MD			✓	D+/+
**HD**	96	Surgical	54	C5/C6	No	9.0	SD			✓	D+/-
**HD**	97	Surgical	46	L5/S1	No	6.0	MD	✓			
**HD**	98	Surgical	65	L3/L4	Yes	6.0	MD	✓			
**HD**	106	Surgical	52	L4/L5	Yes	5.0	MD			✓	D+/-
**HD**	110	Surgical	24	L3	No	3.0	ND			✓	ND-/-
**HD**	111	Surgical	68	L4/L5	No	8.0	SD			✓	D-/-
**HD**	112	Surgical	42	L5/S1	No	11.0	SD			✓	D-/-
**HD**	124	Surgical	40	L4/L5	No	3.5	ND			✓	ND-/-
**HD**	127	Surgical	27	L5/S1	No				✓		
**HD**	145	Surgical	38	L4/L5	No	8.15	SD	✓			
**HD**	146	Surgical	47	L5/S1		5.85	MD	✓			
**HD**	148	Surgical	70	C5/C6		6.0		✓			
**HD**	151	Surgical	47	L5/S1		5.3	MD	✓			
**HD**	153	Surgical	30	L5/S1	No	6.0	MD	✓			
**HD**	154	Surgical	52	L4/L5	No	6.0	MD	✓			
**HD**	156	Surgical	24	L4/L5	Yes	3.0	ND	✓			
**HD**	157	Surgical	31	L5/S1	No	5.0	I	✓		✓	D+/+
**HD**	158	Surgical	38	L4/L5	No	7.0	I	✓	✓		
**HD**	159	Surgical	39	L4/L5	No	3.0	I	✓			
**HD**	160	Surgical	26	C3/C5		6.0	MD	✓			
**HD**	166	Surgical	33	L4/L5	Yes	8.0	SD	✓			
**HD**	170	Surgical		L5/S1	No	8.0	SD	✓	✓		

(*Continued*)

Reference		Source	Age	IVD Level	IVD Intact?	Average Grade	Classification	DE	Cytokine Treatments	IHC	Cohort
**HD**	174	Surgical	29	L5/S1	Yes	7.0	MD	✓			
**HD**	175	Surgical	37	L5/S1	Yes	8.0	SD	✓			
**HD**	177	Surgical	51	L4/L5		2.0	N				
**HD**	182	Surgical	35	L5/S1	Yes	5.0	I				
**HD**	184	Surgical	49	L4/L5		8.0	SD	✓			
**HD**	187	Surgical			Yes	4.0	ND			✓	ND-/-
**HD**	192	Surgical	29	L5/S1	No	8.0	SD	✓			
**HD**	194	Surgical	35	L4/L5	No	11.0	SD	✓			
**HD**	195	Surgical	38	C6/C7	No	9.0	SD	✓			
**HD**	197	Surgical	42	L4/L5	No	8.0	SD	✓			
**HD**	207	Surgical	38	L5/S1	No	7.0	MD			✓	D+/-
**HD**	219	Surgical	54	L5/S1	No	10.5	I	✓			
**HD**	225	Surgical	36	L5/S1	Yes	9.0	SD			✓	D-/-
**HD**	228	Surgical				6.0	I	✓			
**HD**	229	Surgical	56	L5	Yes	6.0	MD	✓			
**HD**	231	Surgical	56	L4/L5	No	5.0	MD	✓		✓	D-/-
**HD**	232	Surgical	58	L5/S1	No	8.0	SD	✓			
**HD**	233	Surgical	44	L5/S1	Yes	9.5	SD	✓			
**HD**	234	Surgical	54	L5/S1	Yes	8.5	SD	✓			
**HD**	243	Surgical	28	L4/L5	Yes	6.0	I	✓		✓	D+/+
**HD**	245	Surgical	24	L4/L5	Yes	4.0	ND			✓	ND-/-
**HD**	246	Surgical	40	L5/S1	Yes	7.5	I	✓			
**HD**	253	Surgical	63	C5/C6	Yes	5.0	I	✓		✓	D+/-
**HD**	254	Surgical	47	L4/L5	No	7.0	I	✓			
**HD**	257	Surgical	46	L5/S1	No	4.5	I	✓			
**HD**	272	Surgical	27	L5/S1	No	4.5	ND			✓	D+/-
**HD**	276	Surgical	71	C5/C6		6.0	MD	✓		✓	D-/-
**HD**	281	Surgical	27	L4/L5	Yes	3.0	ND	✓			
**HD**	E13-00073	PM (UT)	28	L4/L5		5	MD			✓	D+/-
**HD**	507-164	PM (UT)	45	L4/L5		5	MD			✓	D-/-
**HD**	507-001	PM (UT)	80	L4/L5		6	MD			✓	D+/-
**HD**	507-234	PM (UT)	80	L4/L5		6	MD			✓	D+/-

(*Continued*)

Reference		Source	Age	IVD Level	IVD Intact?	Average Grade	Classification	DE	Cytokine Treatments	IHC	Cohort
**HD**	506-219	PM (UT)	54	L4/L5		7	MD			✓	D-/-
**HD**	506-145	PM (UT)	59	L4/L5		7	MD			✓	D+/-
**HD**	507-110	PM (UT)	63	L4/L5		8	SD			✓	D+/-
**HD**	506-290	PM (UT)	88	L4/L5		9	SD			✓	D+/-
**HD**	506-136	PM (UT)	75	L4/L5		9	SD			✓	D+/+
**HD**	508-070	PM (UT)	21	L4/L5		9	SD			✓	D+/-
**HD**	504-134	PM (UT)	-	L4/L5		9	SD			✓	D+/+
**HD**	506-272	PM (UT)	68	L4/L5		10	SD			✓	D+/-
**HD**	507-051	PM (UT)	82	L4/L5		10	SD			✓	D+/+
**HD**	508-263	PM (UT)	28	L4/L5		11	SD			✓	D+/-
**HD**	506-191	PM (UT)	67	L4/L5		11	SD			✓	D-/-
**HD**	507-192	PM (UT)	63	L4/L5		11	SD			✓	D+/+
**HD**	506-179	PM (UT)	59	L4/L5		11	SD			✓	D+/+
**HD**	507-066	PM (UT)	80	L4/L5		12	SD			✓	D+/-

Supplementary Table 2: Target antibodies used for IHC, their optimal concentrations and antigen retrieval methods. Heat antigen retrieval consisted of 10 minute microwave irradiation in 0.05 M Tris buffer, pH 9.5 pre-heated to 60°C. Enzyme antigen retrieval consisted of incubation of tissue in 0.01% w/v chymotrypsin/$CaCl_2$ (Sigma, UK), for 30 minutes at 37°C

Target Antibody	Clonality	Optimal Dilution	Antigen Retrieval Method	Secondary Antibody	Serum Block
**NF200 (ab82259)**	Mouse Monoclonal	1/400	Heat	Rabbit anti mouse	Rabbit
**PGP9.5 (ab8189)**	Mouse Monoclonal	1/200	Heat	Rabbit anti mouse	Rabbit
**CD31 (ab28364)**	Rabbit Polyclonal	1/400	Enzyme	Goat anti Rabbit	Goat
**Sema3C (ab135842)**	Rabbit Polyclonal	1/200	None	Goat anti Rabbit	Goat
**Sema3D (ABIN1386639)**	Rabbit Polyclonal	1/200	Enzyme	Goat anti Rabbit	Goat
**NRP-1 (ab81321)**	Rabbit Monoclonal	1/200	Heat	Goat anti Rabbit	Goat
**NRP-2 (ab185710)**	Rabbit Polyclonal	1/200	Enzyme	Goat anti Rabbit	Goat
**Plexin A1 (ab32960)**	Rabbit Polyclonal	1/200	Enzyme	Goat anti Rabbit	Goat
